# Impact of Plant Community Diversity on Greenhouse Gas Emissions in Riparian Zones

**DOI:** 10.3390/plants13172412

**Published:** 2024-08-29

**Authors:** Guanlin Li, Jiacong Xu, Yi Tang, Yanjiao Wang, Jiabao Lou, Sixuan Xu, Babar Iqbal, Yingnan Li, Daolin Du

**Affiliations:** 1 School of Environment and Safety Engineering, Jiangsu University, Zhenjiang 212013, China; liguanlin@ujs.edu.cn (G.L.); ty12212021@163.com (Y.T.); 17797313436@163.com (Y.W.); zdkhh71yobyobyylyl@163.com (J.L.); xusixuan2022@163.com (S.X.); babar@ujs.edu.cn (B.I.); 2 Jiangsu Collaborative Innovation Center of Technology and Material of Water Treatment, Suzhou University of Science and Technology, Suzhou 215009, China; 3 Department of Environmental Design, Jiangsu University, Zhenjiang 212013, China; 3211814031@stmail.ujs.edu.cn (J.X.); 4 Division of Environmental Science and Ecological Engineering, Korea University, Seoul 02841, Republic of Korea; 5 Jingjiang College, Jiangsu University, Zhenjiang 212013, China; 6 Institute of Environment and Ecology, School of Environment and Safety Engineering, Jiangsu University, Zhenjiang 212013, China; 7 School of Emergency Management, Jiangsu University, Zhenjiang 212013, China; 8 School of Agricultural Engineering, Jiangsu University, Zhenjiang 212013, China

**Keywords:** global warming potential, plant community structure, plant species richness, landscape diversity

## Abstract

Plant community succession can impact greenhouse gas (GHG) emissions from the soil by altering the soil carbon and nitrogen cycles. However, the effects of community landscape diversity on soil GHG emissions have rarely been fully understood. Therefore, this study investigated how plant landscape diversity, structure type, and species composition, affect soil GHG emissions in a riparian zone. Soil GHG emissions were assessed by measuring the air samples collected from four study sites, which have different plant community structure types and species compositions (natural sites with complex plants, landscaped sites with fruit trees and grasses, untended sites with ruderals, and farmland sites), using the static chamber method. Significant differences were observed in soil carbon dioxide (CO_2_; *p* < 0.001), nitrous oxide (N_2_O; *p* < 0.001), and methane (CH_4_; *p* = 0.005) emissions. The untended site with ruderals exhibited the highest CO_2_ emissions, while N_2_O emissions increased as plant community diversity decreased. All sites acted as sinks for CH_4_ emissions, with decreased CH_4_ uptake efficiency in more diverse plant communities. The Mantel test and variance partitioning analysis revealed soil microbial biomass as an indirect influencer of GHG emissions. This study could help predict soil GHG emissions and their global warming potential under future changes in the island riparian zones.

## 1. Introduction

Owing to the increase in atmospheric concentrations of greenhouse gases (GHGs), the average global surface temperature continues to rise, leading to global warming and affects the carbon and nitrogen cycles. Carbon dioxide (CO_2_), nitrous oxide (N_2_O), and methane (CH_4_) are the major GHGs, contributing to approximately 60%, 6%, and 20% of the global warming potential, respectively [1,2]. Recent studies have reported that the effects of alterations in the diversity of plant communities on GHG emissions from soil can be of a similar magnitude to those of other global change factors, such as warming, land use change, and biological invasion [3,4,5]. Alterations in plant community landscape diversity (such as community structure and species richness) can impact the dynamics of carbon and nutrients in terrestrial ecosystems, thereby shifting the exchange rates of GHGs between land and atmosphere [6]. Thus, developing a mechanistic framework to accurately estimate the GHG emissions under plant landscape diversities is crucial for predicting carbon and nitrogen dynamics, as well as mitigating global warming.

Plant communities can impact soil GHG emissions directly by controlling the substrate (including litter, residues, and root exudates) input into the soil, and indirectly by shaping the microbial community structure and functions [7]. Differences in species composition and community structure result in varying root biomass and organizational components, which can further alter net primary production and the quantity, quality, and components of organic matter inputs, thereby affecting soil GHG emissions [1]. Specifically, soil CO_2_ emissions are primarily attributed to rhizosphere respiration, and CH_4_ is emitted from the decomposition of plant litter and residues [8,9,10]. Moreover, soil parameters (such as soil organic matter content, and microbial activity and functions), are closely linked with CO_2_ and CH_4_ emissions. For instance, it was shown that soil organic carbon content directly correlated with microbial carbon use efficiency, thereby impacting the CO_2_ and CH_4_ emissions from soil [11,12,13]. N_2_O emissions, associated with both nitrification and denitrification processes, are also regulated by the quantity and quality of soil organic matter, as it provides electrons for these processes [8,14,15,16]. Additionally, measured soil parameters like nitrate concentration and redox potential have been shown to directly influence N_2_O emissions [6,17].

Another possible way plant communities affect GHG emissions, particularly through denitrification and methanogenesis processes, is by altering oxygen conditions via oxygen transport through their roots and aerenchymous tissues [18]. Plant species composition and community structure can shape the soil microbial activity, community composition, and functions, and then with consequences on GHG emissions [19,20,21]. In addition, soil microbes also play a crucial role in GHG emissions, as they are key drivers of ecological processes related to GHG emissions, such as mineralization, methanogenesis, nitrification, and denitrification [22,23,24]. Therefore, changes in the landscape diversity of plant communities can substantially affect the dynamics of GHGs in the atmosphere–vegetation–soil system. However, the mechanisms by which plant community landscape diversity influences GHG emissions remain unclear and may depending on environmental conditions. It has been reported that a high diversity of plant communities could positively, neutrally, or negatively impact GHG emissions [14,25,26,27,28]. This suggests that plant community landscape diversity may have the potential to alter GHG emissions through a range of mechanisms that can be reasonably predicted and tested.

The riparian zone, as an ecologically sensitive area and a border between terrestrial and aquatic ecosystems, is characterized by the coexistence and/or alternate presence of natural soils, saturated soils, and standing water, and it is crucial for GHG emissions, carbon sequestration, and nitrogen cycle [2,8,29,30]. The landscape of plant communities along the riparian zones is highly subjected to destruction and gradually diversifies due to anthropogenic activities such as agricultural reclamation, landscape transformation, fishing, and felling. Based on the literature, the quantity and quality of carbon inputs from the plant community may be key factors influencing GHG emissions from soil [22,30,31]. Consequently, variations in plant community diversity can result in changes to plant-derived carbon inputs, ultimately affecting soil GHG emissions [28,32]. To accurately quantify GHG emissions under varying land use and landscape conditions, various methods and models have been employed. For instance, the Before–After–Control–Impact (BACI) model, process-based models, and static chamber methods have proven effective in evaluating the impacts of land use changes and other interventions on GHG emissions in such ecologically sensitive zones. However, there are some limitations to these models and methods, for example, the BACI model may not fully capture the complex interactions between plant community diversity and soil GHG emission processes in riparian zones due to some specific interventions. Other approaches, such as process-based models, are often limited by the availability of detailed input data and thus may not adequately represent the influence of plant community composition and structure on GHG dynamics. Hence, there is still a lack of a mechanistic framework to understand how changes in plant landscape diversity affect GHG emissions from the riparian zone of soil.

Thus, the objectives of the present study were (1) to investigate the effects of plant landscape diversity, considering both structure and species compositions of plant community, on GHG (CO_2_, N_2_O, and CH_4_) emissions from soil; and (2) to quantify the drivers of GHG emissions from a riparian zone of the soil. These findings will advance the current knowledge on GHG dynamics under different plant community landscape diversities.

## 2. Materials and Methods

### 2.1. Study Site

The present study was conducted on Jiangxin Island (32°12′32″ N, 119°35′35″ E) in Zhenjiang City, China. Jiangxin Island, a village located on the Yangtze River, has a subtropical monsoon climate, with a long-term (1991–2020) average air temperature of 12.5 °C and annual precipitation of 1148.7 mm. In recent years, it has been developed as a national agricultural ecological tourism island, owing to its natural beauty, pastoral scenery, and plentiful wetland resources. A river running through the island serves as both the main landscape feature and primary water source for the living and agriculture of the islanders. With the development of the island, the riparian zone of the river was also developed or renovated. Some parts of the riparian zone have been landscaped with different plant communities, some have been converted into farmland, and some have retained their natural status quo.

On 12 July 2022, 5 × 5 m plots were established along the riparian zone of the river, encompassing four different plant community landscape types, with three replicates for each type. The minimum distance between neighboring plots was 10 m. The four plant community landscapes were natural plant community sites (Site 1) with structurally complex plants (arbor, shrub, and grasses), landscaped sites (Site 2) with orange trees and grasses, untended or abandoned sites (Site 3) with ruderal, and farmland sites (Site 4) with crops. The plant communities at each site were investigated, and the diversity indices, including the Abundance-based Coverage Estimator (ACE) and Evenness, were calculated using the equations described by Wilsey et al. (2007) [33].

### 2.2. Greenhouse Gas (GHG) Emission and Global Warming Potential Measurement

In this study, the GHG emissions from each plot were measured by applying static chamber and gas chromatography techniques. The static chamber was customized using PVC with a length × width × height of 30 × 30 × 30 cm. A fan, a balloon, and a temperature sensor were installed inside the static chamber to mix the air, stabilize the air pressure, and measure the temperature. The gas samples were collected on 17 July 2022. In brief, three customized chambers were inserted into the soil along the diagonal line of each plot after removing the ground plants. The gas samples at 0, 15, 30, and 45 min after chamber enclosure were collected and stored in sealed bags. The temperature was recorded at each sampling time point. The concentrations of carbon dioxide (CO_2_), nitrous oxide (N_2_O), and methane (CH_4_) in each sample were determined using gas chromatography (Agilent 7890, Agilent Technologies Inc., Wilmington, DE, USA). The emissions of CO_2_ (mg C m^−2^ h^−1^), N_2_O (mg N m^−2^ h^−1^), and CH_4_ (mg C m^−2^ h^−1^) were calculated using the equations described by Cheng et al. (2007) [34] and Hu et al. (2022) [30].

Global warming potential is widely used to quantify GHG inferences on global warming, calculated as the sum of GHG (CO_2_, N_2_O, and CH_4_) emissions from the soil. Utilizing CO_2_ as the reference gas, the global warming potential was by converting CH_4_ and N_2_O emissions into CO_2_ equivalents. Based on a 100-year timeframe, the conversion coefficients for CH_4_ and N_2_O emissions to CO_2_ equivalents were 28 and 265, respectively [35]. The global warming potential (mg CO_2_ equivalent m^−2^ h^−1^) was calculated by applying the equation described by Hsieh et al. (2021) [7].

### 2.3. Soil Samples Collection and Soil Parameters Measurement

Soil samples were collected on 17 July 2022, following gas sample collection. In each plot, topsoil samples (0–15 cm depth) were taken from the three chamber positions using a soil corer (2.4 cm diameter). After passing through a sieve (2 mm) to remove stones and visible plant debris, the soil samples were divided into two portions, one of which was air-dried for soil pH, dissolved organic carbon (DOC), dissolved organic nitrogen (DON) content, and available phosphorus (SAP) content analyses. And the other portion was stored at 4 °C for soil moisture (SM), inorganic nitrogen (IN) content, microbial biomass carbon (MBC) content, microbial biomass nitrogen (MBN) content, microbial biomass phosphorus (MBP) content, microbial extracellular carbon-acquisition enzyme activity (EEA_C_), microbial extracellular nitrogen-acquisition enzyme activity (EEA_N_), microbial extracellular phosphorus-acquisition enzyme activity (EEA_P_), and microbial energy (VL) and nutrient limitation (VA) analyses. In particular, IN was determined by summing the contents of nitrate nitrogen and ammonium nitrogen. In addition, EEA_C_ and EEA_N_ were determined by summing the activities of β-1,4-glucosidase, β-D-1,4-cellulobiohydrolase, and β-1,4-xylosidase and the activities of β-1,4-N-acetylglucosaminidase and L-leucine aminopeptidase, respectively. All the soil parameter measurements and calculation methods are described in the previous study [31,36,37,38]. Additionally, the stoichiometric ratios of DOC to DON (DO_C:N_), DOC to SAP (DO_C:P_), DON to SAP (DO_N:P_), MBC to MBN (MB_C:N_), MBC to MBP (MB_C:P_), and MBN to MBP (MB_N:P_) were calculated.

### 2.4. Statistical Analysis

Significant differences in plant community diversity parameters, soil parameters, GHG emissions, and global warming potential among the plant community landscape sites were tested using a one-way analysis of variance (ANOVA) followed by Fisher’s least significant difference test at *p* < 0.05. Principal component analysis (PCA) was performed to identify differences in soil parameters and GHG variables. Correlations between the soil parameters and GHG variables were tested using the Mantel test and random forest analysis. Based on the results of the Mantel test and random forest analysis, variance partitioning analysis (VPA) was performed to evaluate the relative importance of soil parameters in the variations in GHG variables. The partial least squares path model (PLS-PM) was used to establish the possible influence of plant community diversity on soil GHG emissions. All analyses were performed using R software version 4.1.1 [39].

## 3. Results

### 3.1. Plant Community Diversity and Soil Parameters

**Figure 1:**
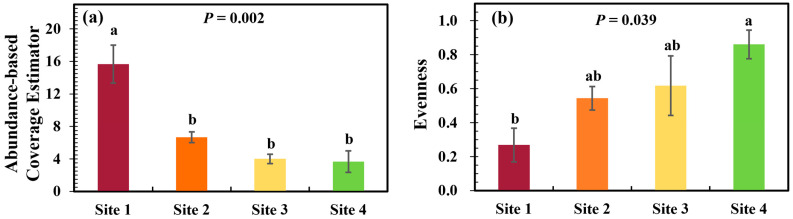
Variations in Abundance-based Coverage Estimator (**a**) and Evenness (**b**) of plant community in each site (n = 3). Site 1: natural plant community sites with structurally complex plants (arbor, shrub, and grasses); Site 2: landscaped sites with orange trees and grasses; Site 3: untended or abandoned sites with ruderal; Site 4: farmland sites with crops. Vertical bars showed the standard error in each site. Different letters denote significant differences at the 0.05 level among the study sites.

Both ACE (*p* = 0.002) and Evenness (*p* = 0.039) diversity indices varied significantly among the study sites. The values of ACE and Evenness from highest to lowest were Site 1 > Site 2 > Site 3 > Site 4 and Site 4 > Site 3 > Site 2 > Site 1, respectively. The opposite trends of ACE and Evenness jointly indicated that plant community diversity was in descending order from Site 1 to Site 2 (Figure 1). Structurally complex plant communities with trees, shrubs, and grasses, including *Ginkgo biloba* L., *Lagerstroemia indica* L., *Osmanthus* sp., *Aesculus chinensis* Bunge, *Ficus religiosa* L., *Citrus reticulata* Blanco, *Acalypha australis* L., *Gynostemma pentaphyllum* (Thunb.) Makino, *Eleusine indica* (L.) Gaertn., *Setaria viridis* (L.) Beauv., *Digitaria sanguinalis* (L.) Scop., *Erigeron canadensis* L., *Metaplexis japonica* (Thunb.) Makino, *Artemisia selengensis* Turcz. ex Bess., *Broussonetia papyrifera* (L.) Vent., *Sonchus wightianus* DC., *Erigeron bonariensis* L., *Bidens pilosa* L., *Erigeron acer* Linn. were observed at Site 1. Orange trees and grasses, including *Citrus reticulata* Blanco, *Acalypha australis* L., *Eleusine indica* (L.) Gaertn., *Setaria viridis* (L.) Beauv., *Portulaca oleracea* L., *Digitaria sanguinalis* (L.) Scop., *Commelina communis* L., *Cucurbita pepo* L., *Maqui berry*, *Gynostemma pentaphyllum* (Thunb.) Makino, *Erigeron canadensis* L., *Amaranthus lividus* L., *Macleaya cordata* (Willd.) R.Br., *Calystegia hederacea* were observed at Site 2. Only abundant ruderals, including *Cynodon dactylon* (L.) Pers., *Setaria viridis* (L.) Beauv., *Maqui berry*, *Artemisia argyi* Levl. et Van, *Amaranthus lividus* L., *Eleusine indica* (L.) Gaertn. were observed at Site 3. Additionally, a farmland site (Site 4) with *Zea mays* L., *Digitaria sanguinalis* (L.) Scop., *Portulaca oleracea* L., *Xanthium strumarium* L., *Erigeron canadensis* L., *Gynostemma pentaphyllum* (Thunb.) Makino, *Cirsium japonicum* Fisch. ex DC., *Calystegia hederacea* were observed.

**Figure 2:**
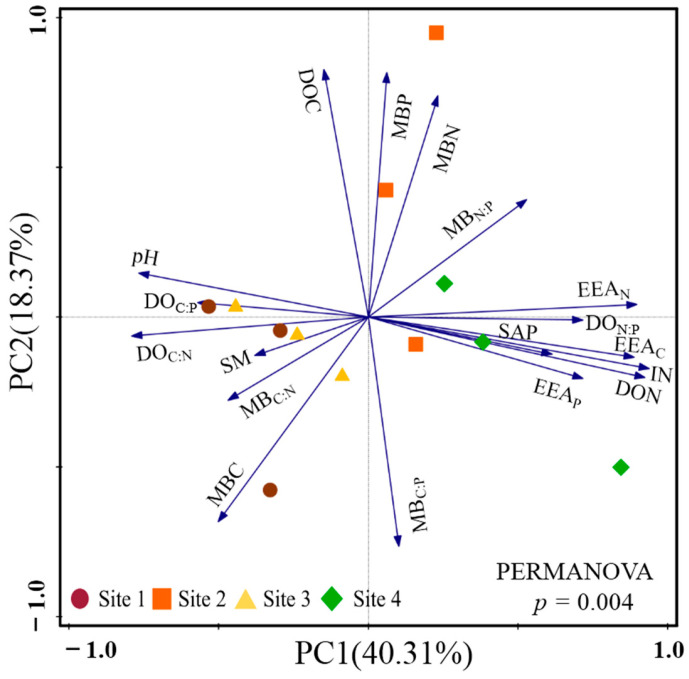
The results of principal component analysis based on the soil parameters among the study sites.

The results of PCA showed that two-dimensional PCA explained 40.31% (PC1) and 18.37% (PC2) of the total variance in soil parameters among the study sites. There was a significant difference in GHG variables among the study sites along the PC1 axis (*p* = 0.003; Figure 2). The SM varied significantly among the study sites (*p* < 0.001), and the highest SM was observed at Site 3. However, there were significant differences in MBC and MBN (all *p* > 0.050). Abnormally higher MBC and MBN were observed at sites 1 and 2, which induced greater variations in the ratio of MBC to MBN among the study sites. VA at all study sites was lower than 45° and varied from 3.40° to 5.78° (*p* < 0.001), which indicated that the soil microbial community in the study sites suffered from nitrogen limitation. In addition, the highest VA was observed at Site 1, whereas the lowest was observed at Site 4 (Table 1).

### 3.2. Soil GHG Emissions and Global Warming Potential

**Figure 3:**
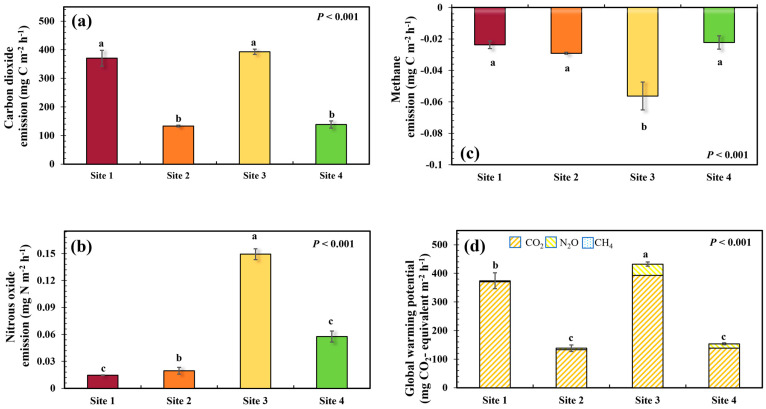
Variations in soil carbon dioxide emission (**a**), nitrous oxide emission (**b**), methane emission (**c**), and global warming potential (**d**) at each site (n = 3). Vertical bars showed the standard error at each site. Different letters denote significant differences at the *p* < 0.050 level among the study sites.

CO_2_ emissions varied significantly among the study sites (*p* < 0.001) and ranged from 133.11 to 392.83 mg C m^−2^ h^−1^. The observed CO_2_ emissions at Site 3 (392.82 ± 9.17 mg C m^−2^ h^−1^) and Site 1 (370.34 ± 27.65 mg C m^−2^ h^−1^) were almost 3 times greater than those at Site 4 (138.44 ± 3.17 mg C m^−2^ h^−1^) and Site 2 (133.11 ± 12.51 mg C m^−2^ h^−1^; Figure 3a).

Significant differences were also observed in the N_2_O emissions among the study sites. N_2_O emissions oscillated between 0.01 to 0.14 mg N m^−2^ h^−1^. The highest value was observed at Site 4 which was 2.60–10.26 times higher than that of the other sites (Figure 3b).

Soils in the present study functioned as the sinks of CH_4_ emissions, the emissions varied from −0.06 to −0.02 mg C m^−2^ h^−1^ with significant differences among all sites (*p* = 0.005). The highest CH_4_ sink was observed at Site 3, whereas the lowest was observed at Sites 1 and 4 (Figure 3c).

In the present study, the global warming potential was exhibited by the GHG emissions from each study site, which was calculated by converting N_2_O and CH_4_ to CO_2_ equivalents. Similar to the individual gases, total GHG emissions also varied significantly among the study sites (*p* < 0.001), ranging from 137.38 to 431.08 mg CO_2_ equivalent m^−2^ h^−1^. It is worth noting that CO_2_ emissions contributed 90.19% to 99.32% of the total GHG emissions, resulting in the variation trends of GHGs at each site being closely aligned with those of CO_2_ emissions. In detail, GHG emissions at Site 3 (431.08 ± 7.59 mg CO_2_ equivalent m^−2^ h^−1^) and Site 1 (373.01 ± 28.20 mg CO_2_ equivalent m^−2^ h^−1^) were approximately 2.72–3.16 times greater than those at Site 4 (153.15 ± 3.49 mg CO_2_ equivalent m^−2^ h^−1^) and Site 2 (137.38 ± 11.92 mg CO_2_ equivalent m^−2^ h^−1^; Figure 3d).

**Figure 4:**
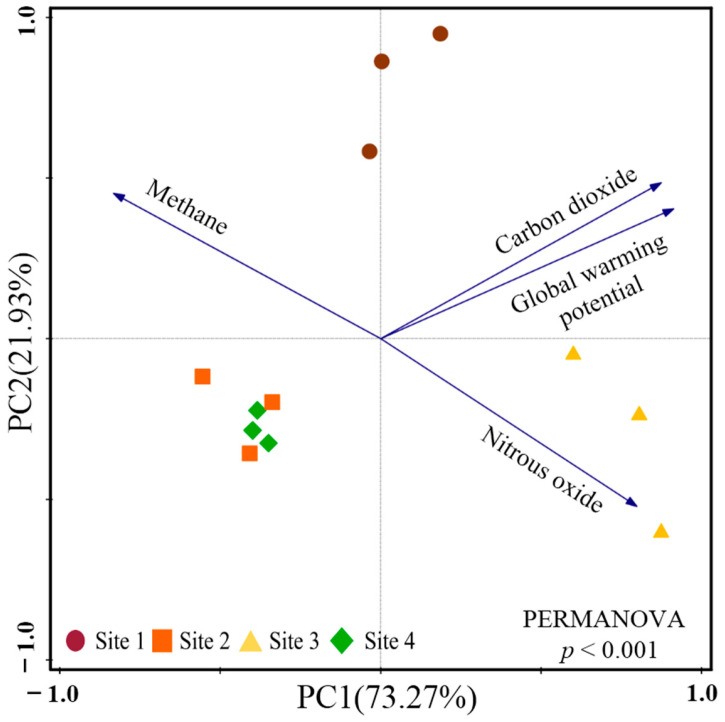
The results of principal component analysis based on soil carbon dioxide emission, nitrous oxide emission, methane emission, and global warming potential among the study sites.

In addition, the results of PCA showed that the first two principal components explained 73.27% (PC1) and 21.93% (PC2) of the total variance of GHG variables among the study sites. There was a significant difference in GHG variables among the study sites along the PC1 axis (*p* = 0.011; Figure 4).

### 3.3. Response of Soil GHG Emissions to Soil Parameter

**Figure 5:**
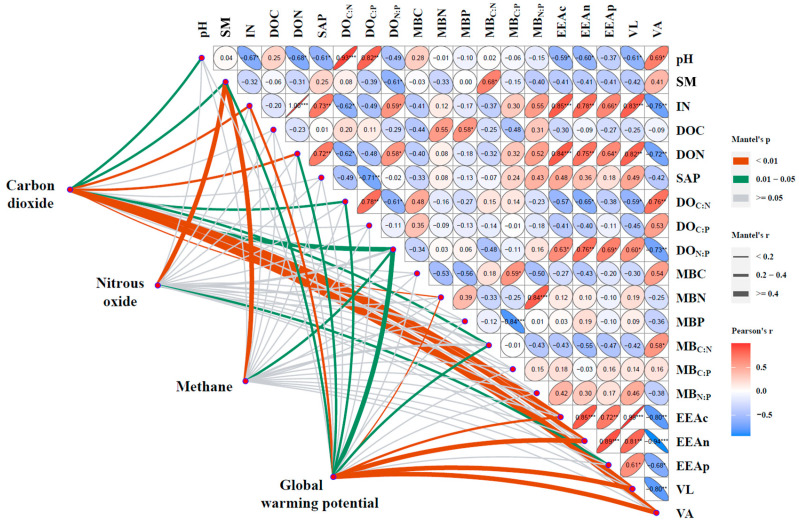
The Mantel tests based on correlations between soil carbon dioxide emission, nitrous oxide emission, methane emission, global warming potential variables, and soil parameters. The grey line means no significance (*p* > 0.050); the green line means significance (0.050 > *p* > 0.010); the red line means significance (*p* < 0.010); the width of the line indicates the strength of the different correlations. The red and blue ovals indicate the strength of the correlation between the two indicators. Red represents positive correlation, and blue represents negative correlation. The correlation between environment variables was obtained by Pearson algorithm, and the correlation between response variables and environment variables was obtained by Mantel algorithm. * = significant at the level of *p* < 0.050, ** = significant at the level of *p* < 0.010, and *** = significant at the level of *p* < 0.001.

**Figure 6:**
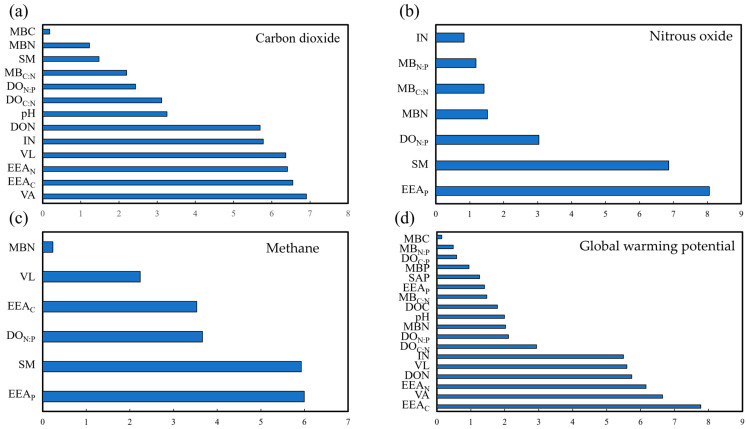
The random forest analysis based on correlations between soil carbon dioxide emission (**a**), nitrous oxide emission (**b**), methane emission (**c**), and global warming potential (**d**) variables and soil parameters.

The Mantel test was based on correlations between soil parameters and GHG variables. The Mantel test revealed that the GHG variables were significantly related to the soil parameters across the total samples. Specifically, GHG variables were significantly correlated with pH, SM, IN, DON, DO_C:N_, DO_N:P_, MBN, MB_C:N_, EEA_C_, EEA_N_, VL, and VA in the soil (Figure 5). Moreover, random forest analysis identified soil microbial parameters, as well as microbial metabolism as the most important predictive factors for the impact of plant community landscape diversity on soil CO_2_ (6.91%, Figure 6a), N_2_O (8.06%, Figure 6b), CH_4_ emissions (5.99%, Figure 6c), and global warming potential (7.77%, Figure 6d).

**Figure 7:**
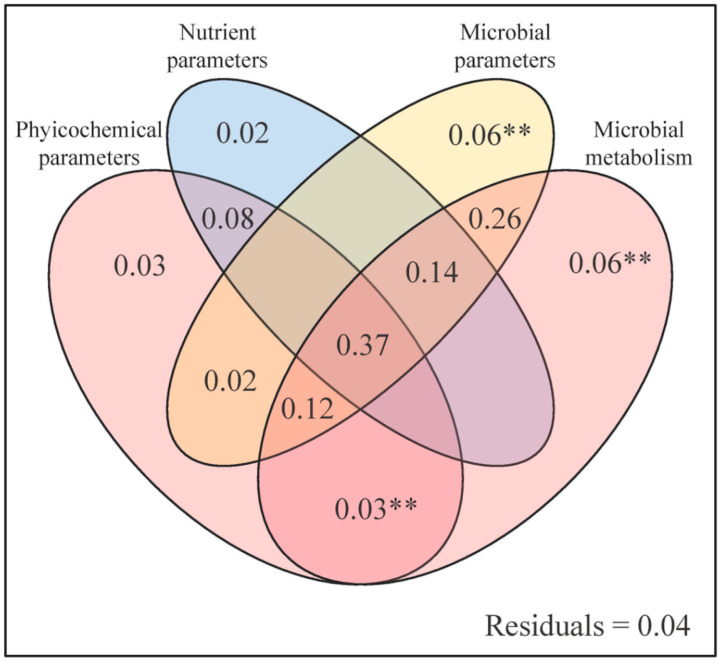
Results of variation partitioning analysis showing the effects of the soil physicochemical parameters, nutrient parameters, microbial parameters, and microbial metabolism parameters on soil carbon dioxide emission, nitrous oxide emission, methane emission, and global warming potential variables. Physicochemical parameters include pH and SM; nutrient parameters include IN, DOC, and DO_C:N_; microbial parameters include EEA_P_, EEA_N_, and MB_C:N_; microbial metabolism includes VL and VA. ** = significant at the level of *p* < 0.010.

Based on the results of the variance partitioning analysis, soil microbial parameters (EEA_N_, EEA_P_, and MB_C:N_) and microbial metabolism (VL and VA) have significant effects on GHG variables among the study sites. In addition, the interaction between physicochemical parameters (pH and SM) and microbial metabolism also has a significant influence on GHG emissions (Figure 7).

### 3.4. Influence Path of Plant Community Diversity on Soil GHG Emissions

**Figure 8:**
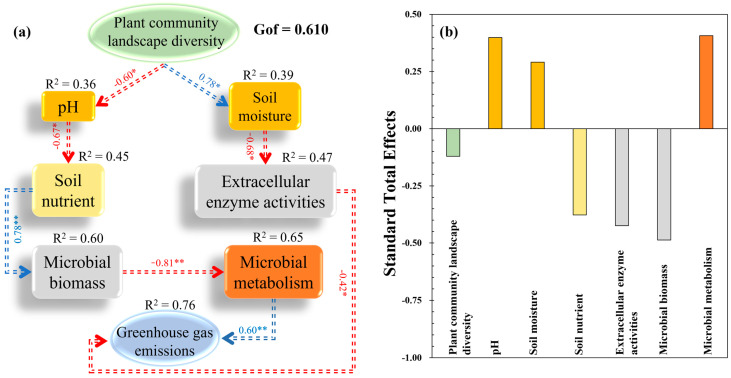
Cascading relationships of the greenhouse gas emissions with soil parameters among the study sites (**a**) and the total effect of each predictive factor on greenhouse gas emissions (**b**). Partial least squares path modeling disentangling major pathways of the influences of soil parameters and plant community landscape diversity on greenhouse gas emissions. Blue and red arrows indicate positive and negative flows of causality. * = significant at the level of *p* < 0.050, and ** = significant at the level of *p* < 0.010.

PLS-PM was established for the path analysis of GHG emissions from soil among the study sites (Gof = 0.610). PLS-PM revealed that differences in plant community diversity at each study site could induce alterations in soil GHG emissions. On the one hand, plant community diversity among the study sites directly affected the soil GHG emissions. On the other hand, plant community diversity affected soil microbial properties, especially microbial metabolism, by altering soil physical properties (i.e., pH and SM) and nutrient properties, thereby ultimately affecting soil GHG emissions (Figure 8a). Microbial biomass (−0.49) was the most influential factor in soil GHG emissions in the present study (Figure 8b).

## 4. Discussion

### 4.1. Direct Effect of Plant Community Landscape Diversity on Soil GHG Emissions

Both the structure and species diversity of plant communities can impact the quantity and quality of carbon inputs into soils [40], which may further alter GHG emissions from the soil. Thus, it was originally hypothesized that different structural types and species compositions of plant communities would induce alterations in soil GHG emissions. As expected, significant differences in GHG emissions were observed among the study sites with various landscape diversity. Specifically, relatively higher soil GHG emissions were observed at Site 3, which is an untended or abandoned ruderal region with low species diversity and a simple community structure, while the lowest was observed at Site 4, a human-transformed agricultural region. In the vegetated regions (i.e., sites 1 to 3), higher plant community diversity tended to reduce N_2_O emissions and CH_4_ uptake, which is in line with the findings of previous studies (Figure 3) [2,28].

Considering that the substrate inputs from plants into the soil were important for GHG emissions and given the marked differences in soil nutrients observed among the study sites in the present study (Figure 5, Figure 6 and Figure 7; Table 1), it was inferred that the difference in variation, quantity, and quality of plant substrate inputs may essentially affect GHG emissions from soils [9,30,41]. The GHG emissions in the agricultural region (Site 4) were the lowest, this may be due to the lower abundance of plants and the reduced availability of substrates used for GHG production (Figure 3). 

Additionally, roots play a crucial role in regulating GHG emissions in several ways. On the one hand, roots could provide channels for transporting GHGs and contribute to CO_2_ emissions through their respiration activity [42]. At this point, the number and variety of plants may be the reason for higher GHG emissions, specifically CO_2_, observed in the regions with more diversified vertical structures of plant communities (Tree–Shrub–Grass structure regions; Site 1) and ruderal regions (Site 3). On the other hand, diversified plant communities with high biomass production could reduce N_2_O emissions by decreasing nitrate nitrogen in the soils [28]. In addition, plant roots can release oxygen into the soil, reoxidizing alternative electron acceptors, such as sulfate, thereby inhibiting CH_4_ production [42]. Furthermore, the density and architecture of roots can also cause changes in soil aeration, and the deposition and utilization patterns of carbon and nitrogen [6]. 

### 4.2. Plant Community Landscape Diversity Altered GHG Emissions through Changing Soil Parameters

Soil moisture, nutrient availability, and the microbial community are considered key factors that influence both abiotic and biotic processes affecting GHG emissions [30,31,43,44,45,46]. Since these abiotic and biotic factors are closely associated with the plant community, changes in plant community landscape diversity would inevitably alter GHG emissions from soil, which was confirmed again in the present study [47,48]. 

Soil moisture is a crucial factor that can both directly and indirectly influence GHG emissions, due to their effects on soil microbial activity, nutrient availability, and physical properties. The observed differences in SM among the diverse plant community landscape regions may be one of the possible reasons for the induced differences in soil GHG emissions. Indeed, a strong correlation between SM and GHG emissions was observed in this study (Figure 5 and Figure 6). This is evidenced by the PLS-PM that the effects on soil GHG emissions were regulated by the impact of plant community landscape diversity on SM, which further altered soil available nutrients and microbes (Figure 8). Hence, the differences in SM across the study sites, particularly the higher SM levels observed at Site 3, may have contributed to the higher CO_2_ emissions and reduced CH_4_ uptake, as well as the variations in N_2_O emissions. Moreover, SM can dissolve CO_2_ and impact the soil’s physical structure (e.g., porosity and aggregate), thereby altering the transportation of GHGs [49]. In addition, SM could control the composition, structure, and activity of microbial communities by altering the diffusion and availability of oxygen and soluble nutrients in the soil, thereby altering the production of GHGs [44,50,51,52]. With the variation in SM, the soil oxygen content changes as well, which will probably influence the quantity and activity of functional microbes such as methanotrophic bacteria and denitrifying bacteria, consequently impacting the ecological process (e.g., denitrification, nitrification, and methanogenesis) and production of GHGs [20,21].

Soils can act as either sinks or sources of CH_4_, with the balance between these roles being regulated by methanogenesis and CH_4_ oxidation. In the present study, no net CH_4_ emissions were observed; instead, the soils functioned as a CH4 sink (Figure 3), suggesting that CH_4_ oxidation consistently outweighed methanogenesis. When oxygen availability is limited in soils, methanogenesis can prime the activity of methanotrophic bacteria to consume and utilize CH_4_ as an energy source, thereby increasing the uptake of CH_4_ from the atmosphere [6]. Thus, at Site 3, the untended ruderal region exhibited the highest SM content, which likely facilitated increased microbial respiration and promoted anaerobic conditions. This, in turn, could reduce CH_4_ oxidation and enhance CO_2_ emissions. In contrast, the agricultural Site 4 had lower SM, which may have limited microbial activity, resulting in lower CO_2_ emissions and a different GHG emission profile. The differences in SM in different plant community landscape diversity regions could further indicate a soil moisture-independent effect on GHG emissions, which could explain the observed pattern.

In addition, plants can affect GHG emissions by altering the quantity and quality of available nutrients in the soil either by secreting root exudates or by competing with other plants or microorganisms. In this study, significant differences in nutrient availability were observed among the study sites (Table 1), which could be easily affected by SM, especially IN and DOC, thereby further regulating the production of GHGs (Figure 5 and Figure 8). This may be linked to that the bioavailable carbon and nutrient sources, such as IN and DOC, can serve as critical substrates for microbial respiration, carbon mineralization processes, and nitrification and denitrification processes, altering the production of GHGs [22,30,53,54]. In general, more diverse plant communities have higher IN capture efficiency and result in relatively lower IN accumulation efficiency, which could sequentially affect denitrification and nitrification, as well as their associated emissions and dynamics of N_2_O [6]. Thus, the N_2_O emissions in Site 1 were lower than others (Figure 3).

Changes in the structure and type of plant community could induce shifts in the activity and function of soil microbial communities [55,56]. The soil moisture-mediated effects on microbial biomass were shown by PLS-PM results, which also revealed that soil microbial metabolic limitation was the main driving force for GHG emissions. That is, the GHG emissions increased with microbial metabolism (VA; Figure 8). Meanwhile, the Mantel test and VPA results also evidenced that microbial biomass (MBC and MBN) indirectly influenced GHG emissions (Figure 5 and Figure 6), which was in line with a previous finding that GHG emissions increased with microbial biomass and activity [57]. MBC and MBN not only reflect the biomass, activity, and element amount of the microbial community in the soil but also regulate the microbial energy and/or nutrient requirements [58]. Thus, MBC, MBN, and their ratios have the potential to operate as metabolic substrates for microbial communities, thereby impacting the production and emissions of GHGs.

### 4.3. Implications for Managing Plant Community Diversity to Mitigate Soil GHG Emissions in Riparian Zones

The findings of this study indicated the role of plant community landscape diversity in modulating soil GHG emissions in riparian zones. The observed differences in GHG emissions among various plant community structures highlight the critical influence of vegetation diversity on soil carbon and nitrogen dynamics. Specifically, areas with low plant community diversity, such as untended ruderal regions (Site 3), exhibited higher CO_2_ emissions and global warming potential (Figure 3). This suggests that landscape management practices aimed at enhancing plant diversity in these regions could potentially mitigate climate change impacts. Furthermore, the study also highlights the importance of soil microbial communities in mediating GHG emissions. The positive correlation between MBC and MBN with GHG emissions indicates that microbial activity is a crucial driver of soil GHG emissions (Figure 5 and Figure 7). Therefore, strategies aimed at optimizing microbial community composition and activity through vegetation management might influence soil GHG dynamics. These findings contribute to a deeper mechanistic understanding of how plant community diversity and soil microbial processes interact to regulate GHG emissions.

However, it is important to acknowledge the potential limitations of generalizing the findings from the specific riparian zone of Jiangxin Island to other regions with different climates, seasons, soil, or vegetation conditions. Future studies are necessary to include comparative analyses across different geographical areas, seasons, and soils, as well as specific root traits or plant–microbial interactions. This will be helpful to assess the consistency of the patterns observed in this study and to develop a more generalized understanding of the interactions between plant community diversity and soil GHG emissions. Such insights are essential for developing predictive models of GHG emissions under various land use and climate change scenarios. Moreover, the results emphasize the need for integrated landscape management approaches that consider both above-ground biodiversity and below-ground microbial processes to effectively mitigate GHG emissions and enhance carbon sequestration in riparian ecosystems.

## 5. Conclusions

In this study, significant differences in soil GHG emissions were observed among sites with diverse plant community landscape diversity, which demonstrated that plant community landscape diversity can potentially alter GHG emissions from the soil. Specifically, the untended or abandoned regions with ruderal tended to exhibit the highest CO_2_ emissions and global warming potential. Soil N_2_O emissions increased with the reduction in the plant community landscape diversity in vegetation regions. The soils at the studied sites functioned as CH_4_ sinks, with decreased CH_4_ uptake efficiency observed as plant community landscape diversity declined. The response of GHGs to changes in plant community landscape diversity may depend on the variations in root structure and function, the quantity and quality of plant inputs, and associated changes in moisture and microbial communities in the soil. Therefore, analyzing the impacts of the landscape diversity-dependent plant communities on GHG emissions from the soil in riparian zones is crucial. While these findings shed light on the impact of vegetation diversity on GHG emissions, they may vary under different climatic, season, soil, and vegetation conditions. Future research is needed to encompass a broader range of climates, seasons, soil types, and vegetation structures to validate and compare the applicability of these findings. This will not only enhance the public understanding of the mechanisms driving GHG emissions but also provide a more comprehensive basis for vegetation landscape management in riparian zones.

## Figures and Tables

**Table 1:** Soil parameters among study sites, presented as means ± standard errors (n = 3).

Parameters	F	*p*	Site 1	Site 2	Site 3	Site 4
pH	2.7	ns	8.30 ± 0.05 a	8.18 ± 0.01 ab	8.21 ± 0.05 ab	8.15 ± 0.02 b
SM (*w*/*w*%)	10.02	**	0.21 ± 0.01 b	0.20 ± 0.01 b	0.24 ± 0.01 a	0.21 ± 0.01 b
IN (×10^−1^ μg^−1^ N g soil)	4.28	*	0.54 ± 0.07 b	6.08 ± 0.44 ab	2.53 ± 1.25 b	11.63 ± 4.51 a
DOC (×10^−1^ mg C g^−1^ soil)	1.33	ns	0.25 ± 0.03	0.32 ± 0.04	0.27 ± 0.01	0.24 ± 0.025
DON (mg N g^−1^ soil)	3.65	ns	0.04 ± 0.01	0.24 ± 0.01	0.11 ± 0.06	0.46 ± 0.18
SAP (mg P g^−1^ soil)	2.12	ns	0.03 ± 0.01	0.10 ± 0.02	0.13 ± 0.03	0.13 ± 0.06
DO_C:N_	3.57	ns	7.27 ± 2.70	1.33 ± 0.22	4.19 ± 1.64	0.71 ± 0.26
DO_C:P_	4.81	*	10.13 ± 3.18 a	3.29 ± 0.36 b	2.22 ± 0.54 b	2.60 ± 1.06 b
DO_N:P_	5.39	*	1.55 ± 0.48 bc	2.76 ± 0.84 ab	0.79 ± 0.37 c	3.52 ± 0.17 a
MBC (×10^−1^ mg C g^−1^ soil)	2.98	ns	0.83 ± 0.10	0.53 ± 0.08	0.66 ± 0.06	0.59 ± 0.053
MBN (×10^−1^ mg N g^−1^ soil)	1.03	ns	0.03 ± 0.02	0.37 ± 0.33	0.02 ± 0.02	0.06 ± 0.02
MBP (×10^−1^ mg P g^−1^ soil)	1.44	ns	0.03 ± 0.01	0.07 ± 0.01	0.04 ± 0.01	0.04 ± 0.02
MB_C:N_ (×10^−1^)	3.2	ns	3.75 ± 1.44	0.93 ± 0.45	8.84 ± 3.82	1.12 ± 0.30
MB_C:P_ (×10^−1^)	0.96	ns	3.22 ± 1.29	0.96 ± 0.41	1.64 ± 0.31	2.45 ± 1.43
MB_N:P_	0.66	ns	1.13 ± 0.51	4.99 ± 4.07	0.68 ± 0.57	3.66 ± 2.91
EEA_C_ (×10^−3^ nmol h^−1^ g^−1^ soil)	7.1	*	2.75 ± 1.33 b	4.55 ± 2.06 b	2.65 ± 1.46 b	7.26 ± 1.59 a
EEA_N_ (×10^−2^ nmol h^−1^ g^−1^ soil)	414.21	***	2.94 ± 0.02 c	4.31 ± 0.07 b	2.93 ± 0.12 c	6.05 ± 0.04 a
EEA_P_ (×10^−1^ nmol h^−1^ g^−1^ soil)	129.1	***	2.97 ± 0.02 b	2.93 ± 0.01 b	2.80 ± 0.02 c	3.59 ± 0.05 a
VL (×10^−2^)	5.02	*	0.93 ± 0.05 a	1.56 ± 0.06 ab	0.95 ± 0.05 b	2.04 ± 0.47 a
VA (°)	101.59	***	5.78 ± 0.09 a	3.88 ± 0.08 ab	5.45 ± 0.19 b	3.40 ± 0.07 a

Different letters denote significant differences (*p* < 0.05) among study sites. ns means no significance, * means significant level *p* < 0.050, ** means significant level *p* < 0.010, and *** means significant level *p* < 0.001.

## Data Availability

Dataset available on request from the authors.
